# Malicious Activity Detection in Lightweight Wearable and IoT Devices Using Signal Stitching

**DOI:** 10.3390/s21103408

**Published:** 2021-05-13

**Authors:** Fatih Karabacak, Umit Ogras, Sule Ozev

**Affiliations:** 1 School of Electrical, Computer, and Energy Engineering, Arizona State University, Tempe, AZ 85281, USA; Sule.Ozev@asu.edu; 2 Department of Electrical and Computer Engineering, University of Wisconsin-Madison, Madison, WI 53706, USA; uogras@wisc.edu

**Keywords:** hardware/firmware trojan detection, wearable electronic device security, flexible electronic security, IoT security, malicious activity detection, side-channel analysis, spectrum analysis, self-referenced trojan detection

## Abstract

The integrated circuit (IC) manufacturing process involves many players, from chip/board design and fabrication to firmware design and installation. In today’s global supply chain, any of these steps are prone to interference from rogue players, creating a security risk. Therefore, manufactured devices need to be verified to perform only their intended operations since it is not economically feasible to control the supply chain and use only trusted facilities. This paper presents a detection technique for malicious activity that can stem from hardware or firmware Trojans. The proposed technique relies on (i) repetitious side-channel sample collection of the active device, (ii) time-domain stitching, and (iii) frequency domain analysis. Since finding a trusted sample is generally impractical, the proposed technique is based on self-referencing to remove the effects of environmental or device-to-device variation in the frequency domain. We first observe that the power spectrum of the Trojan activity is confined to a low-frequency band. Then, we exploit this fact to achieve self-referencing using signal detection theory. The proposed technique’s effectiveness is demonstrated through experiments on a wearable electronics prototype and system-on-chip (SoC) under a variety of practical scenarios. Experimental results show the proposed detection technique enables a high overall detection coverage for malicious activities of varying types with 0.8 s monitoring time overhead, which is negligible.

## 1. Introduction

The Internet of Things (IoT) compromises a network of computing devices ranging from low-power edge nodes, such as sensors, to more powerful and capable computing systems. For instance, the use of wearable devices has been increasing very rapidly in health applications, such as remote monitoring and diagnosis [1]. Similarly, smart devices are already used for smart grid, smart home, smart city management, and supply chain management. At the same time, the increasing adoption of IoT poses new security challenges, mainly due to its limitations on hardware, compute resources, and power.

Cost and development time are two major problems faced by the designers of low-volume system-on-chips (SoCs). Design reuse has been one of the most effective practices in the semiconductor industry since it can dramatically reduce design, verification, and test costs. Hence, most, if not all, companies employ third-party IP (intellectual property) cores from dozens of vendors to amortize their cost and shrink the design turn-around time. Moreover, the boards and firmware for these devices can also be developed and installed by third-party vendors. With a global supply chain with many players, ensuring the security of wearable and IoT devices is a daunting challenge. Thus, it has become necessary to trust but verify the IoT devices both at production time and in the field [2].

Small form-factor battery-powered embedded systems, such as wearable devices, have limited computing power and battery capacity. These limitations make existing cybersecurity mechanisms such as anti-virus software and anomaly detectors [3] too costly to implement. Thus, ensuring the security of wearable devices with acceptable overhead is a new challenge that requires cost-conscious solutions. Security of embedded devices becomes even more critical because the increased inter-connectivity provides more space for attackers to introduce the Trojans [4].

Attacks on wearable devices can be performed using Trojans implemented as malicious hardware or firmware modifications. The wide range of attack space has resulted in exponentially increasing security problems. To evade detection, the malicious modifications are hidden carefully with minimal overhead, and they activate either randomly or after specific trigger incidents [5]. Therefore, the symptoms of the Trojan are not always observable. Moreover, when a Trojan is active, its impact on measurable factors, such as system performance or power consumption, is typically negligible due to the subtle modification it makes. Detecting hardware and firmware Trojans is challenging due to several factors. First, there is a wide range of trigger mechanisms and payload, which are hard to enumerate. Second, the diversity of intellectual property (IP) cores and their sources amplifies the detection complexity. Finally, one cannot compare the compromised chip against a trusted sample because obtaining a golden source may not be possible. As a result, maintaining the authenticity of each resource is becoming more challenging.

This paper presents a method to detect malicious activity in lightweight wearable and IoT devices. The proposed approach does not rely on any trusted sample; instead, it establishes a baseline for each individual device based on its periodic steady state (PSS) behavior. By referencing the detection threshold to baseline characteristics of each individual device, environment and process variations can be removed. In order to collect data, without affecting operation of the device, the proposed technique uses a signal stitching technique in which side channel information of repetitious code sequences is sampled and processed to monitor device activity.

**Figure 1:**
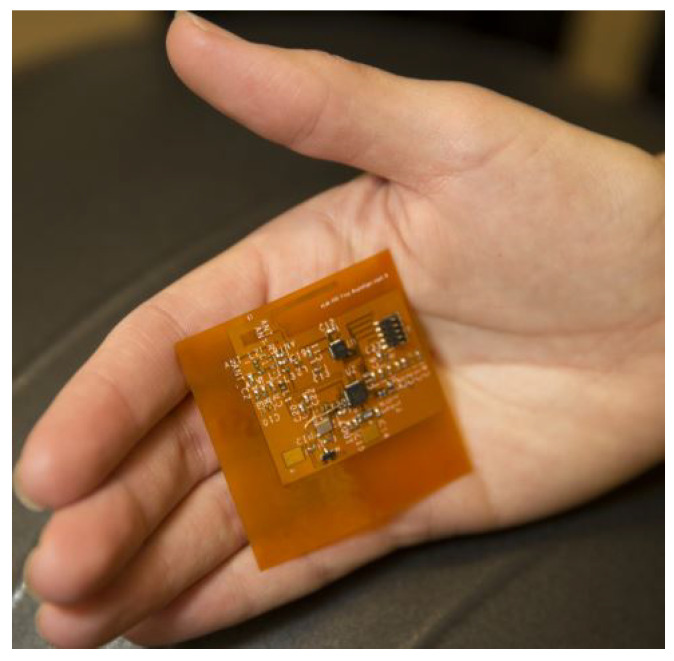
Wearable electronics prototype.

**Figure 2:**
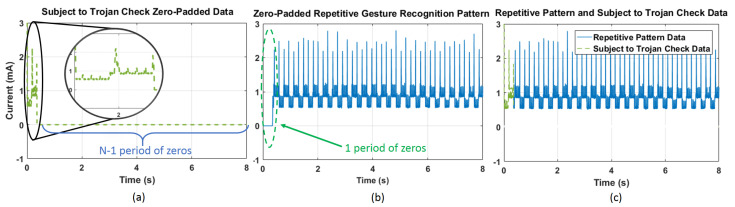
(**a**) Zero padded data that is subjected to Trojan check; (**b**) zero-padded repetitive gesture recognition pattern with one period of zero padding at the starting; (**c**) stitched one period of data to repetitive gesture recognition data.

The proposed technique is demonstrated on the wearable device prototype shown in Figure 1, which runs gesture recognition software including an arbitrary repetitive gesture recognition algorithm. It first brings a side-channel signal (in our case, the power consumption) of the device into a periodic steady-state (PSS). Then, the repetitive patterns are stitched with any existing data from the same device to construct a representative measurement. As an example, Figure 2a shows the run-time data collected from the device in one instance with zero padding. These data are stitched to pre-existing data (collected earlier) shown in Figure 2b, to obtain the complete data sequence, shown in Figure 2c. The complete data can be analyzed in the frequency domain to establish criteria for flagging suspicious activity. The collected side channel data can be expressed as the sum of the primary system response (i.e., the power consumption without a Trojan), the Trojan activity, and environment and measurement noise. Placing the device into a PSS concentrates the known application signal power at a specific frequency and its harmonics. This leaves a large portion of the signal spectrum unoccupied and available for detection. If there is a Trojan activity, it will be present over a wider frequency band since it is unlikely to be correlated with primary activity. Thus, the unoccupied bins of the spectrum can be analyzed to determine whether there is unauthorized activity.

The major contributions of this paper are as follows:

- A methodology for time-domain signal switching to collect side channel signal information on repetitive primary activity to reduce test duration,
- A limited-bin spectral analysis technique for detecting unauthorized activity to reduce the computational burden of the detection technique,
- A self-referenced malicious activity detection technique applicable to not only sinusoidal excitation but also to repetitive patterns to remove the process and environmental variation effects,
- Evaluation of the proposed approach while running gesture recognition and Wi-Fi applications without requiring a trusted sample.
- Extensive experiments with a wearable electronics prototype [6] and a commercial multiprocessor system-on-chip (MpSoC) [7], and show the effectiveness of the proposed detection technique.

The rest of the paper is organized as follows. The threat model is explained in Section 2. Related work is presented in Section 3. The proposed malicious activity detection technique is described in Section 4. Finally, extensive experimental evaluation is presented in Section 5, and conclusions appear in Section 6.

## 2. Threat Model

Many IoT devices run their own operating system and applications. As these devices become more common, they will also become bigger targets for hackers. Trojans could target the firmware to insert malicious code and gain access to sensitive information or cause damage. They could take the form of passive hardware entities which help malicious software bypass pre-existing hardware protection systems.

**Figure 3:**
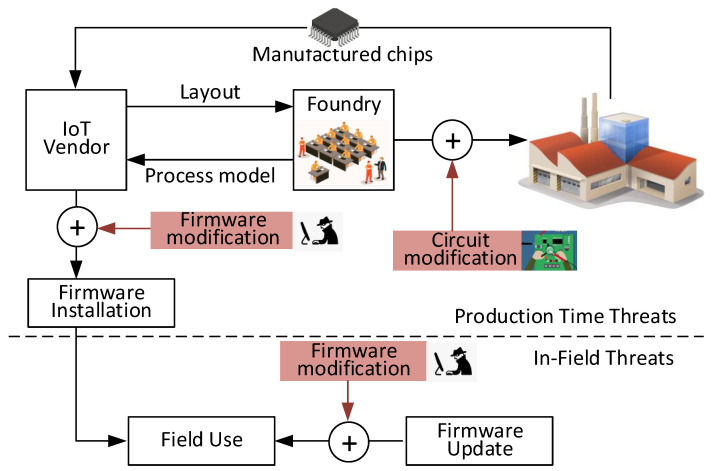
Threat model.

Figure 3 illustrates attack models for potential hardware and firmware Trojan threats. The IoT vendor receives the process model from the foundry and produces the circuit layout using this process model. Malicious modifications can be added at production time or while the device is in use in the field. The threats can originate at the foundry, during firmware design, or production firmware installation. There can be circuit modifications and firmware modifications. Hardware threats originate at the foundry from malicious attackers (e.g., third-party consultants, rogue employees) who modify the hardware to insert malicious circuitry. When triggered by prespecified analog or digital conditions, the modifications can have passive effects, such as leaking information [8], or cause malfunction (e.g., by heating the device). The analog trigger mechanisms include temperature, device aging, or signal delay between two points [9]. Similarly, digital conditions can be implemented as combinational or sequential circuits [10]. Firmware threats can be added during firmware installation at the IoT vendor or other third-party company. The attacker can also make firmware changes during field updates. These changes can require physical contact to the device [11] or done remotely through any network connectivity, such as Bluetooth and Wi-Fi. Majority of firmware updates are delivered via the internet, opening doors to the attackers to invade the device remotely [12]. Hence, every product in the field needs periodic monitoring to verify recent changes made to the system.

## 3. Related Work

Battery-driven embedded systems have limited computing power and battery capacity. These constraints only worsen when the system is subject to demands of security. Embedded devices are attractive targets for today’s sophisticated and innovative attacks since they are not suitable for traditional security mechanisms, such as anti-virus and anomaly detectors, built for general-purpose computers. These attacks can be realized as hardware [13,14,15,16] and firmware modifications [17], known as Trojans. This wide range of attack space has resulted in exponentially increasing security problems.

Hardware Trojans are small-scale circuits designed to perform a malicious operation not intended by the original system [18]. Attackers can insert them at multiple points in the supply chain, such as the foundry [19] or a third-party IP provider [20], as illustrated in Figure 3. Various techniques have been developed to detect hardware Trojans during testing or at run-time in the field [21]. The technique we proposed in this paper falls into the run-time detection category. Run-time hardware Trojan detection approaches can modify the target structure they aim to protect [22]. Similarly, they can employ multiple modular redundancies at the software level to perform a task even with malicious hardware [23]. Test-time approaches employ functional verification [22] and side channel measurements [24,25]. Functional verification techniques use the test patterns with the highest probability of identifying Trojans that cause device logic failure. The side-channel measurement-based techniques attempt to infer a Trojan’s presence by measuring parameters that the Trojan can alter. Path delays, transient supply currents, and average supply currents are examples of these parameters. The difficulty of finding a known reference is a significant challenge for detecting Trojans by side-channel analysis. Typically, this reference is established by a transistor-level simulation [26]. Alternatively, the reference can rely on reliable samples of the circuitry, which may not even exist. Even if a reliable reference can be established, noise in the system and measurement errors present another challenge. Generally, the Trojan symptom (in terms of current/power and delay variance) may also be comparable to predicted variations due to process and environmental noise. In contrast to previous work, our proposed approaches are focused on signal processing with limited-bin spectral analysis and signal detection theory, which eliminates the impact of process and environmental variations. They do not require a golden reference as a benchmark.

Compared to hardware, the firmware is more easily distributed, making it a much easier attack target to compromise the embedded systems. Since embedded devices run on firmware, we need to understand how the firmware works. The firmware provides necessary information for the hardware device to communicate with other devices. Firmware is found on all kinds of computer hardware but is most vulnerable in embedded devices that generate or exchange vast amounts of privacy-sensitive, or security-critical information [27]. Due to increasing demand for connected embedded devices on the emerging IoT [28], firmware security has become more critical than ever to organizations such as banks, governments, and businesses [29,30]. We can classify firmware attacks as static or dynamic. Static firmware attacks focus on modifying firmware code residing in memory via hardware modification or firmware updates or patches [31,32,33]. In contrast, dynamic firmware attacks attempt to exploit dynamic memory components such as stacks and heaps to change the behavior of the firmware control flow [34]. The vulnerabilities leveraged by firmware for malicious modification have been addressed in several research studies ranging from battery-powered personal health monitor devices to conventional industrial control systems [35,36,37]. On the detection and identification side, however, there is limited research work available. Here, detection approaches can be divided as signature based (looking for signatures of known attacks) or anomaly based [38] (modeling the expected behavior of firmware and detecting deviations from this reference model). A recent study focused on a low-cost technique to detect malicious firmware modification in embedded devices by using readily available registers [39]. The proposed framework needs exhaustive offline profiling to generate a reference database. Moreover, this detection mechanism relies on write-protected memory components, which are still vulnerable to alteration through hardware modification. The authors of [40] described a firmware vulnerability in a network adapter by which a remote attacker on the network can gain full access to the victim’s machine. They proposed a pragmatic detection technique that detects any unexpected changes in the control flow when a return value is modified in the network adapter [41]. The work presented in [3] proposes anomaly analysis for embedded firmware by employing source code instrumentation techniques. Any deviation from the referenced run of the firmware is flagged as anomalous. The proposed technique needs to establish a reference model and running the instrumented firmware offline. However, it requires considerable overhead for the computationally intensive task.

Our proposed technique fills a gap in the ability to detect malicious modification. To compare with our previous work [2], in this paper, we propose a self-referenced malicious activity detection technique applicable to not only sinusoidal excitation, but also to repetitive patterns to remove the effects of process and environmental variations. This paper demonstrates a technique to place the device under test in a repetitive state using functional algorithms so as to limit the frequency response of its authorized activity signature in a small number of frequency bins. This allows us to enable detection in the field. The previous work [2] requires specific and dedicated test sequence to achieve repetitive state and resulting energy is more concentrated. However, achieving this repetitive state in the field is difficult. We also propose a methodology for time-domain signal switching to collect side-channel signal information on repetitious primary activity to reduce the test duration. This allows us to collect data over time without disabling the device for an extended duration of time. The extensive experiments are conducted using a wearable electronics prototype and commercial multiprocessor system-on-chip (MpSoC) with real-life examples such as gesture recognition and Wi-Fi application to demonstrate the effectiveness of the proposed detection technique in real world usage conditions. In [2], the hardware experiments were conducted on the only MpSoC with a synthetic example such as simple matrix multiplication to create sinusoidal wave forms.

## 4. Malicious Activity Detection

### 4.1. Run-Time Testing and Signal Stitching Technique

It is highly desirable to detect hardware or firmware Trojans before chips are deployed, but existing techniques cannot guarantee comprehensive a coverage for all types and sizes of Trojans. If a Trojan attack is introduced after production, such as insertion during a firmware update, or was not detected at production time, in-field activity monitoring and run-time testing can significantly reduce its risk. In exchange for some performance overhead, these approaches can flag the device or disable it upon detection of malicious activity. The challenge, however, is that these testing and monitoring activities, like measuring device current, power consumption or memory usage, should not interfere with normal device operation.

Many IoT devices are active only for short intervals, between which the system is placed into a low-power or sleep mode [42]. Hence, the idle periods can readily be used for test data collection. The same set of applications that run during normal operation can be used for PSS generation in test mode during idle time. Therefore, malicious modification(s) cannot evade detection when testing is performed.

The proposed detection technique uses a run-time signal stitching technique to collect data without interrupting its functionality. The device is monitored, and data are collected during idle time. One can also collect data during active periods. However, the associated overhead can affect its performance since resource-constrained wearable or IoT devices have limited processing capability [43]. The data collection with many different applications can further increase Trojan detection coverage by activating additional hardware and firmware components. These applications can run in a predefined test mode configuration to create periodic steady-state conditions.

As long as the device reaches a periodic steady-state, it does not matter when the data are collected. Hence, we can select any suitable time-span for testing, and the best choice is the idle stage of the device. By collecting data during repetitive patterns and stitching it with existing data, the overall operational pattern of the device can be established. After the entire data sequence is assembled, the proposed detection algorithm can be run to determine whether the collected data contains known or unknown authorized activity. The signal stitching technique also enables collection of data during different times of the day. Hence, the technique is able to detect a Trojan that has a larger bandwidth than the length of a single measurement.

**Figure 4:**
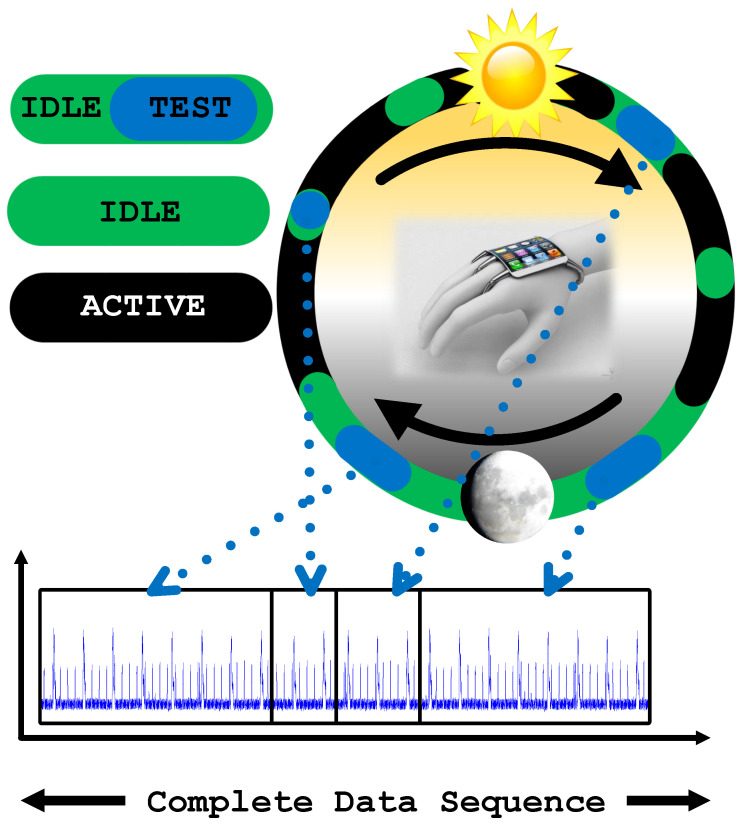
Illustration of device usage in a day and measuring current of device in idle stage for data stitching.

Memory has always been a difficult delicate balance in wearable systems. Unlike personal computers (PCs), tablets, and other devices, wearable devices have significantly smaller memory capacity. However, the memory capacity still must be adequate to support the required functionality and the device firmware. With their expansive growth in popularity, users expect more features and performance from wearable and IoT devices, but these features cannot come at the expense of basic functionality. In other words, improved processing and memory capacity should come with improved security and privacy features. Thus, the memory is a requirement for the proposed malicious activity detection. To this end, performing data collection and analysis during idle times means there will free memory that is sufficient for running the detection algorithm. As seen in Figure 4, the device is monitored and during its idle time, part of the data are measured. This data are stitched with previously collected data samples to synthesize a complete data sequence. When the required data sequence is collected, frequency domain analysis can be run.

### 4.2. Optimized Fast Fourier Transform (FFT) Algorithm for Zero-Padded Data

IoT or wearable devices typically run on limited battery operated power and therefore present unique challenges in terms of availability of computational resources. Thus, a new challenge to ensuring the security of wearable devices is power awareness [44]. Activity monitoring, measurement of complete data sequences, and detection algorithm computation add significant overhead that requires further power aware optimization.

**Figure 5:**
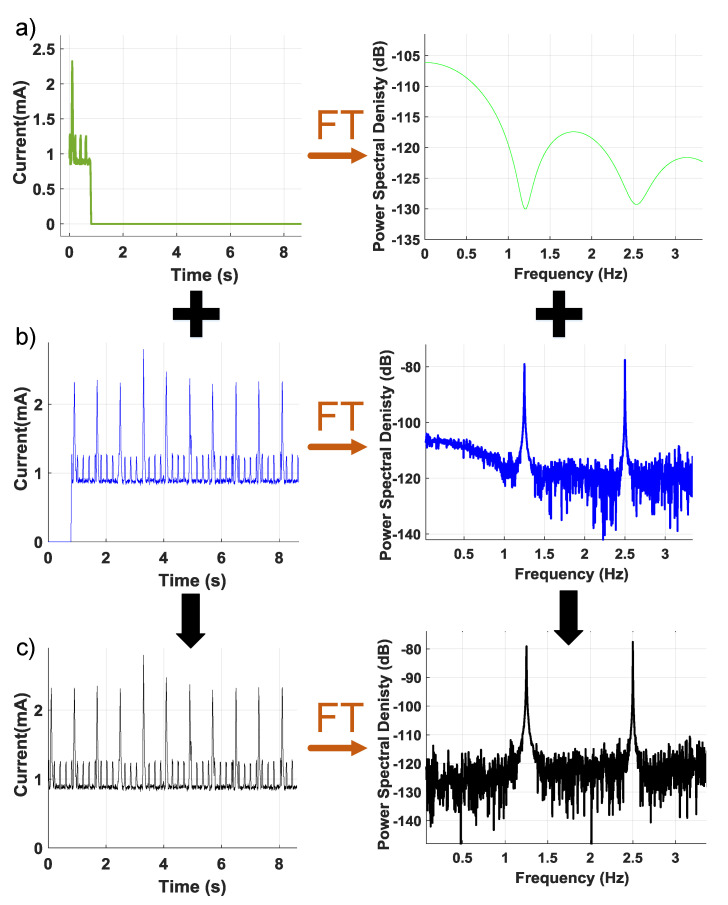
Illustration of superposition of Fourier Transform that is using saved and a period of data to determine suspicious activity. (**a**) One period of data time and frequency response. (**b**) The collected data are zero padded for a period of samples and then its Fourier transform. (**c**) Complete spectrum that can be analyzed for suspicious activity.

First, the proposed detection method relies upon identification of a repetitive pattern in the main application to effectively decouple the fundamental frequency and its harmonics from other unexpected activity. Thus, some monitoring time is required for the detection algorithm to function. However, we can minimize the monitoring time by reusing previously collected and analyzed data which has not been flagged for suspicious activity. Figure 5a shows a shorter period of data that have been collected and zero-padded to match the size of the complete data sequence for Fourier transform analysis. In Figure 5b, the collected data are zero padded for a period of samples and then its Fourier Transform is saved. The Fourier Transform of saved and zero-padded data can be summed as seen in Figure 5c to assemble a complete spectrum that can be analyzed for suspicious activity.

Second, the computation time and power consumption need to be minimized to support a seamless, energy efficient security monitoring system. The Recursive Fast Fourier Transform (FFT) algorithm can be optimized by skipping zero padded data as in Algorithm 1, where Input represents the zero-padded period of measured data, NFFT represents the sample size of the test data, and Size represents the original number of sample points before zero-padding. For optimal efficiency, the number of samples in the stitched data set must be a power of two. The optimized FFT algorithm is 4.2 times faster than the standard recursive FFT algorithm for NFFT = 1024 and Size = 32 samples. We will discuss more about computation time of the FFT in experimental data in Section 5.
**Algorithm 1:** Optimized recursive fast Fourier transform for zero-padded data
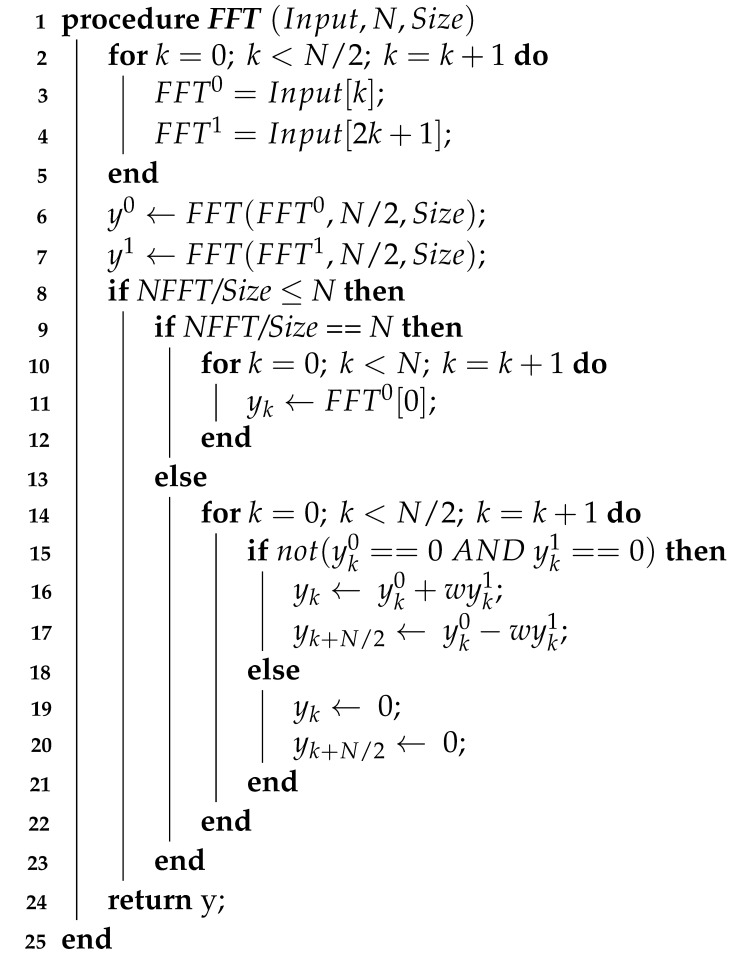


### 4.3. Proposed Detection Technique Overview

The major problem in detecting unauthorized activity is obtaining a golden signature, which is extremely difficult, and in some cases, unfeasible. Moreover, Process-Voltage-Temperature (PVT) variations and environmental noise can mask the effect of the Trojan circuit on measured parameters (e.g., power [45], even if multi-dimensional analysis is used [26,46]). Therefore, a decision on the presence (or absence) of a Trojan should also preferably be taken without the need for a golden or trusted reference.

To detect minor power anomalies due to malicious operation, the primary circuit response must be decoupled from that of the Trojan and any background noise. We achieve this by generating a test sequence such that the *signal* (i.e., the response of the device) has spectral properties that can be differentiated from the Trojan activity. We can denote the measured power P(t) as follows:

(1) $$P\left(t\right)=P_{0}\cdot sin\left(\omega_{0}t\right)+\varepsilon \left(t\right)+n\left(t\right)+P_{Tr}\left(t\right)$$

where $P_{0}\cdot sin\left(\omega_{0}t\right)$ is the sinusoidal power consumption of the primary circuit, ε(t) denotes the error that we make in setting up the sine wave, n(t) is random noise, and $P_{Tr}\left(t\right)$ is the power consumption of the Trojan circuit [2,47,48]. The power spectrum of the primary signal, i.e., $P_{0}sin\left(w_{0}t\right)$ is concentrated in one frequency location. The noise signal has a flat spectral signature. While the specific details of the Trojan activity are unknown, its switching speed is clearly limited by the system clock. It is also unlikely that the Trojan activity period will match with the primary signal periodicity. Therefore, the FFTs of Trojan and primary activity will inevitably occupy different frequency bins. As a result, the Trojan signal bandwidth will be limited, making it different from white noise and the primary signal. The spectrum of P(t) is the sum of the spectra of its components. The primary signal, $P_{0}\cdot sin\left(\omega_{0}t\right)$ is concentrated in one frequency location. The error signal, ε(t), can be modeled as a pulse train:

(2) $$\varepsilon \left(t\right)=\left(\sum_{i=1}^{N_{p}}\varepsilon_{i}\cdot p\left(t-iT_{s}\right)\right)*\left(\sum_{k=1}^{N_{s}}\delta \left(t-kT_{0}\right)\right)$$

where $N_{p}$ is the number of samples in the sine wave, $T_{s}$ is the sampling period, $\varepsilon_{i}$ is the magnitude of the approximation error, p(t) is a pulse with duration $T_{s}$, $N_{s}$ is the number of sine wave periods in the measurement duration, and $T_{0}$ is the period of the sine wave. Note that, while the error of the sine wave approximation within a single period can be random, the error signal itself is also periodic with the same period as the original sine wave. Hence, the error signal’s power spectrum will be concentrated at the harmonics of $f_{0}$, as in Equation (3):

(3) $$S_{\epsilon }\left(f\right)=\sum_{i=1}^{N_{p}}\frac{2\varepsilon_{i}}{N_{s}}\delta \left(f\right)+\sum_{n=1}^{N-1}\left(\sum_{i=1}^{N_{p}}\frac{2\varepsilon_{i}}{n\pi }sin\left(\frac{n\pi }{N_{s}}\right)\right)\delta \left(f-nf_{0}\right)$$

n(t) is the noise signal. Hence, its power will spread through the *entire spectrum*. Finally, $P_{Tr}\left(t\right)$ is the *unknown Trojan signal*.

Although the specific aspects of the Trojan operation are unclear, the system clock explicitly limits its switching speed. We may therefore confidently presume that this is not related to the primary signal. As a result, the bandwidth of the Trojan signal would be limited, rendering it separate from white noise and the main signal.

**Figure 6:**
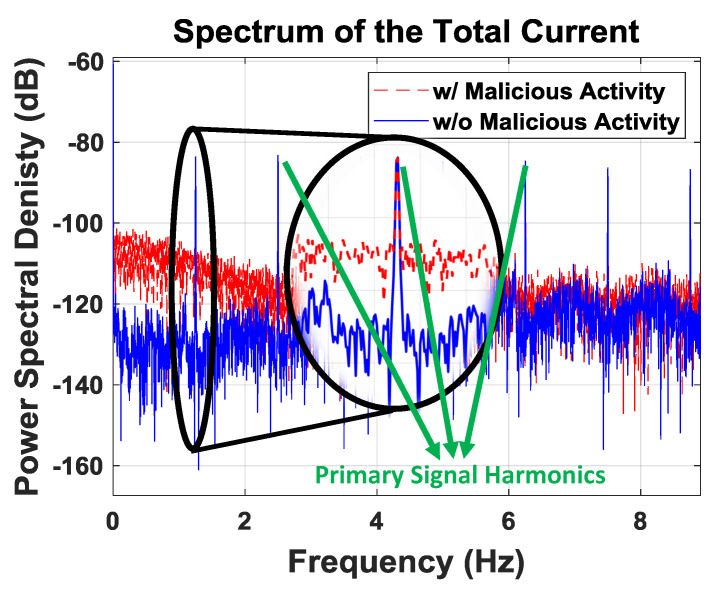
Spectrum of the total current.

Figure 6 shows that the operation of Trojan has a clear fingerprint. However, we cannot say that we have understanding of this continuum, since we do not know how Trojan performs. In order to identify an unexpected signature, we concentrate on what is anticipated of the system and flag if there is any abnormal behavior. This will allow us to decide if the spectrum differs noticeably from its intended state.

The detection algorithm uses a run-time signal stitching technique that eliminates hardware variation, and the self-referencing technique removes the effect of process and environmental variation. It can detect any activity that consumes power. The detection method utilizes a periodic run of the device application(s) to create a steady state that concentrates the known signal power at a known frequency. Moreover, using the native application(s) of the device for steady state characterization allows for normal operation and triggering of the conditions that activate the Trojan. In addition, coverage of triggering conditions can be increased by designing patterns that activate different hardware and firmware portions of the device. In summary, if there is a Trojan that is activated while the application is running, the proposed algorithm is able to detect this abnormal behavior.

**Figure 7:**
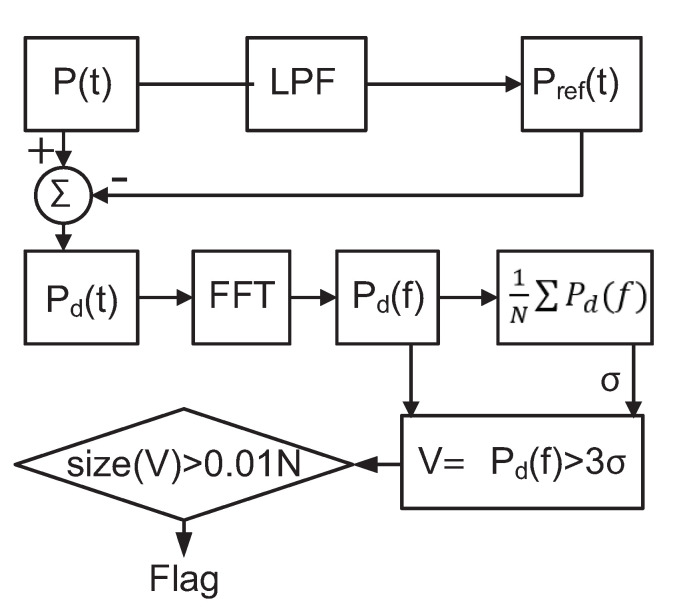
Detection algorithm.

The proposed detection algorithm is shown in Figure 7. We sample and process the composite power signal P(t) in the digital domain. We first pass the power signal through a low-pass filter, whose cut-off frequency is chosen to include harmonics of the primary signal that are above the noise floor. The resulting signal $P_{ref}\left(t\right)$ is subtracted from that of P(t) to exclude primary signal components from the analysis. The resulting residual time-domain signal $P_{d}\left(t\right)$ contains noise, the majority of the Trojan signal energy, and some additional components due to modeling effects. The noise level, $\sigma_{n}$, can be determined from the residual spectrum $P_{d}\left(f\right)$ by taking an average of the spectrum. To avoid false positives due to noise, we set a spectrum threshold of $3\sigma_{n}$ (corresponding to a confidence level of nearly 99.7%), and record spectral components (*V*) exceeding this threshold. We extend the mathematical model to remove the need for sinusoidal excitation, which may not be practical in an Internet of Things (IoT) context. Similarly, there is a need for techniques to detect malicious activity in lower power IoT devices. To this purpose, this paper uses a random repetitive pattern to operate the device under test so that its current consumption is pushed into a PSS. At the same time, the Trojan is excited in an uncorrelated state. In this way, the system’s output is decoupled from that of the Trojan activity and the noise.

(4) $$S_{f}\left(f\right)=\sum_{n=1}^{\infty }\frac{2P_{f}}{n\pi }sin\left(\frac{n\pi d}{T}\right)\delta \left(f-nf_{0}\right)$$

Detection of unknown malicious activity can be generalized to any periodic foreground signal, including a periodic pulse train, as in Equation (4), where $P_{f}$ represents the power of each pulse, *d* represents the duration of each pulse, and *T* represents the period of the pulse train. Hence, it is possible to use this self-referencing technique where the foreground (legitimate) activity is repetitive, resulting in a power signature in the shape of a pulse train. This can be achieved either by intentionally repeating a program segment or identifying a program segment that repeats and adjusting the sampling window accordingly. If the same foreground frame is captured several times, it is possible to obtain a spectral signature of the foreground activity. The spectral signature of each window then is compared to this average to determine whether the signature contains the expected flat spectrum (due to noise) or unknown unauthorized activity in addition to noise which would result in a non-flat spectrum. We use the high-frequency portion of the spectrum as a reference to judge the *flatness* of the resulting signature. By placing a threshold on the spectral signature, we can determine how many bins violate this threshold. The number of such bins should not exceed 1% (due to the 3σ band). Hence, violations beyond this number are treated as evidence of unauthorized malicious activity.

## 5. Experimental Evaluation

### 5.1. Experimental Setup

**Figure 8:**
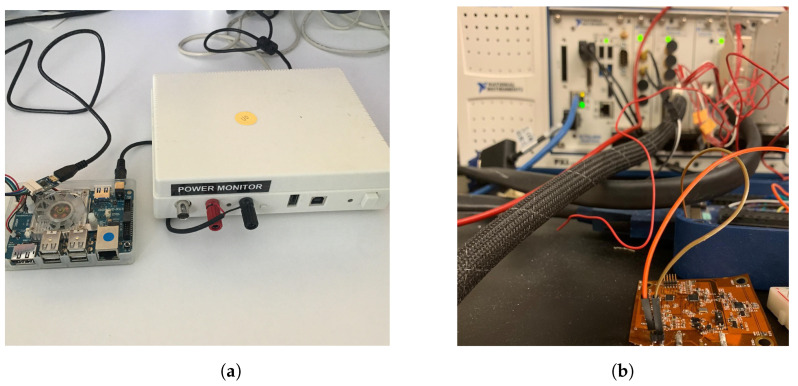
(**a**) Wi-Fi experiment setup with Power monitoring tool. The Odroid board is connected to a low voltage power monitor tool from Monsoon Solution; (**b**) flexible prototype experiment setup with NI-DAQ machine.

To evaluate the proposed approach, we perform two sets of experiments, one on an IoT device prototype [6] and another on a commercial MpSoC [7]. The experiment setup is shown in Figure 8. The wearable IoT device runs a gesture recognition algorithm under a limited energy budget [49] and is representative of low-power IoT devices used for human–computer interaction and mobile health monitoring. The second application scenario uses Wi-Fi communication, which is omnipresent in many mobile application scenarios, ranging from smartphones to simple Wi-Fi enabled IoT devices. In each of these cases, the Trojan activates randomly, resulting in different time/frequency signatures. We performed the experiments at different times during the day to demonstrate that the system does not generate false positives. For the Wi-Fi application, we used C++ code portion to inject firmware Trojan to disturb the system. The foreground application reads sensor information and transmits the data over Wi-Fi to a target destination. To model the malicious activity, we employ a simple firmware Trojan that copies the sensor data to a separate location at random instances.

The wearable and IoT device is capable of performing multiple tasks and is configured to fulfill one or more needs of a specific target group. It can support applications such as gesture recognition, temperature reading, location reporting, heart rate reading, and health/fitness monitoring, all of which create a periodic steady state without requiring any special arrangements during the design stage. We have to remember that an exhaustive testing strategy is universally acknowledged to be prohibitive because designs of even moderate complexity would require hundreds of years to test comprehensively.

These various periodic applications can be selected to reduce test generation effort and will effectively account for continuous working and environmental changes. In addition to these functional applications, dedicated firmware that concentrates the power spectrum on known harmonics can be added and run periodically. Such an application can run at arbitrary frequencies unknown to the attacker, thereby obscuring the test strategy, even if the attacker has knowledge of the design, and changing the run frequency from one test period to another will further help to prevent compromising the detection system. In our implementation, we use gesture recognition and Wi-Fi applications, which are expected to create a repetitive pattern while the device is in use in the environment.

### 5.2. Malicious Activity

When a Trojan is inserted into a chip, it is almost certain that it will consume power. The Trojan’s contribution to the circuit’s overall power consumption, but, on the other side, is highly dependent on its size and type. We know that overall Trojan activation includes functional and structural forms, which would be incredibly difficult given that the Trojan’s size and form are uncertain. A trigger circuit and a payload circuit are common components of Trojans. Using the trigger inputs and/or internal states of the circuit, the trigger circuit determines whether the Trojan payload condition is met or not. The trigger portion is often thought of as always-on circuitry that controls the activity of the compromised device in order for the triggering sequence to take place. The payload circuit creates malfunctions such as leakage of information, downgrading performance of the circuit, or catastrophic failure of the system.

For our analysis, we will consider the output effect of the Trojan on power consumption while the trigger circuit or payload circuit is working. Based on observation of power consumption, we are able to detect abnormalities or malicious activity at the device. Thus, we must be able to separate the components of current that are likely to be consumed by a Trojan in the infested device. In this work, we present four types of Trojans and their energy ratio in Table 1. From the standpoint of the trigger circuit, Trojans appear to increase power usage or unnecessary processes, resulting in faults or a gradual degradation in product efficiency. Since their impact on the regular circuitry is negligible at any given moment, detecting them is difficult.

In an ideal case, our malicious activity detection method applies circuit input vectors, triggers the malicious activity, observes the unintended behavior, and reports it to the design owner. However, in practice, the activation of malicious activity is very difficult and sometimes is impossible, as we do not have enough information about the Trojan’s features, including its location (firmware or hardware), the trigger condition, and the malicious functionality. Therefore, we propose to focus on the side-channel signal of the Trojan as the primary detection method as power consumption of the Trojan is a substantial side-channel signal analysis parameter. In this article, we pay particular attention to determining the smallest detectable Trojan, i.e., the lowest energy that a Trojan may have and still be detected, using one of these four types of malicious activities that are enabled at random times. Instead of focusing on identifying triggering and activation mechanisms, our proposed method is intended to detect a malicious addition of any kind. We should also note that the always-on type of Trojan that shows constant direct current (DC) consumption is not the target of the proposed detection method. This type of Trojan can easily be found by a time domain power trace detection method [50].

### 5.3. Comparisons to Existing Trojan Detection Methods

**Figure 9:**
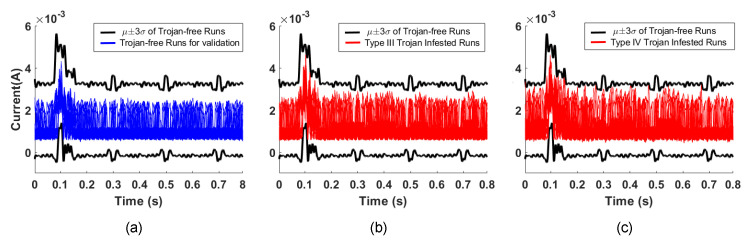
The μ±3σ current consumption envelope of 500 Trojan-free runs and the current consumption of 100 Trojan-free runs (**a**), the current consumption of 100 Type-I Trojan-infested runs (**b**), and the current consumption of 100 Type-II Trojan-infested runs (**c**).

By comparison, time domain analysis of the same current measurement data for our proposed Trojan types cannot distinguish between Trojan-free or Trojan-infested runs. This occurs because the Trojan’s current consumption is hidden within the margins allowed for process and environment variations [51]. In this way, malicious activity can be hidden within the run from the system level examination. Figure 9a plots the current consumption when a period of gesture recognition application is run by 100 of the Trojan-free runs, as well as the μ±3σ envelope of the current consumption for 500 of the Trojan-free runs. Figure 9b,c plot the current consumption when a period of gesture recognition application run by the Type-III and Type-IV Trojan-infested runs, respectively. This demonstrates that the malicious activity is still within the μ±3σ envelope and cannot be detected by time domain analysis.

### 5.4. Proposed Detection Algorithm Optimization

Many Trojan identification proposals investigate the presumption of rarity. The aim is to cut down on the monitoring and computation time of the test (i.e., the size of the collected information from device), while increasing the confidence level that the system being tested is free of Trojans. This is very desirable as it lessens the test time (faster testing is needed for resource-constrained device) and eliminates the stress of continuous testing.

As seen in Table 2, our detection algorithm can be used with a different set of test modes. To use the proposed detection algorithm, different test modes can be selected based on the device usage. We also evaluate the sensitivity of our detection method in the presence of measurement and background noise. Our proposed test requires collection of 20 s of data which can be collected without system interruption for the test Mode 1. When the complete data sequence is collected, the analysis of the data takes approximately 60 s to run with standard recursive FFT algorithm. Since test Mode 1 may not be practical during periods when the device is in heavy use, we apply a signal stitching technique to eliminate the need for continuous data collection. Thus, the monitoring time can be reduced to approximately 0.8 s. Once the full sequence of data are collected, a regular FFT can be run which takes approximately 60 s to complete the analysis for the test Mode 2.

Battery-powered devices have limited resources to support normal operation, so security or monitoring functions need to minimize their resource requirements. The proposed technique consumes 80 s for Mode 1 testing, which can be done during device idle time. The Mode 1 test also represents the most exhaustive method that was explored. As seen in Table 2, other test modes require much less time and achieve acceptable coverage. The computation time of a regular FFT can be further optimized for our proposed detection technique as seen in the test Mode 3. Part of the data from the Fourier Transform is saved, as seen in Figure 5b. A limited period of data are measured and zero-padded to the same size of the complete data sequence. If the Fourier Transform of the zero-padded data are added to the saved Fourier Transform, which was previously analyzed and not flagged for any suspicious activity, we can safely analyze one period of data using our proposed optimized FFT. Hence, the single test computation time will go down approximately 4.2 times with optimized FFT as seen in Table 2. Test Mode 3 is used to analyze a short period of data that was collected recently, while Mode 2 waits until the device collects and stitches a full data sequence for analysis. Test Mode 2 works effectively with data collected throughout the day to detect the abnormalities and provides higher detection coverage. In contrast, test Mode 3 is able to run the detection algorithm based on only a short period of available data.

### 5.5. Gesture Recognition Application

The gesture recognition algorithm runs with a period of 0.8 s on the IoT prototype attached to the user’s wrist [52]. The prototype features test ports to measure power consumption of the microprocessor. The power consumption was measured using NI PXIe-4081 and PXIe-4080 digital multimeter systems with a 5 kHz sampling frequency. During the recognition algorithm running, the motion processing unit records the accelerometer and gyroscope readings. Then, the micro-controller processes the sensor data using a neural network to recognize the user gesture. Finally, the recognized gesture is transmitted via Bluetooth Low Energy (BLE) communication protocol. While the application runs in the foreground, various malicious programs, as seen in Table 1, are launched randomly on the micro-controller.

**Figure 10:**
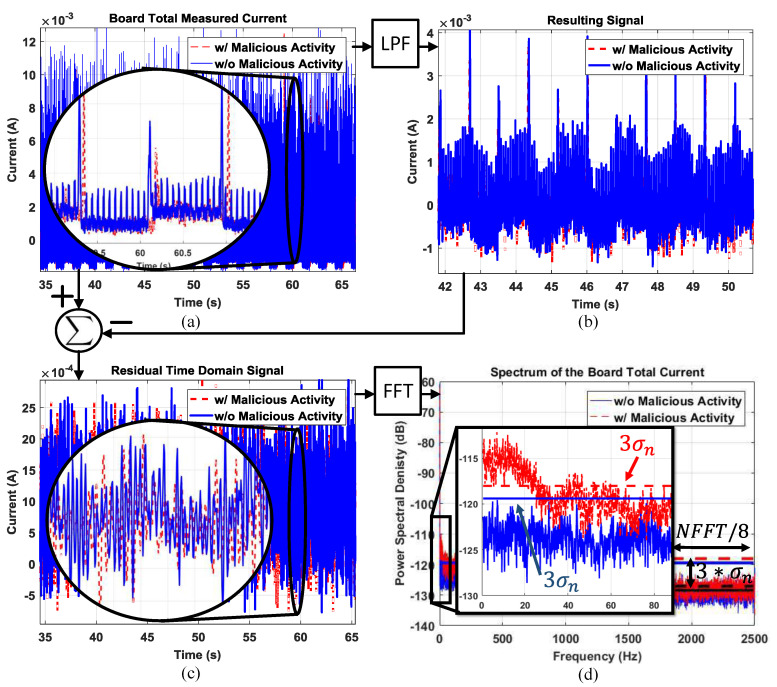
(**a**) The total current drawn by the IoT prototype; (**b**) the current signal after the low-pass filter; (**c**) the resulting residual time domain signal, which contains the noise signal and the majority of the malicious activity energy; (**d**) the residual signal spectrum with calculated noise threshold levels.

The total current drawn by the IoT prototype with and without Trojan activity is depicted in Figure 10a. We observe that the time domain signals are almost identical, highlighted also by the zoomed-in section of the plot. The current signals after low-pass filtering are plotted in Figure 10b. The difference between the original and filtered signal gives the residual signal as shown in Figure 10c. This residual time domain signal contains the noise and the majority of the malicious activity energy, but the malicious activity is still not differentiable. Finally, we provide the power spectrum of the current with and without Trojan activity in Figure 10d. The frequency domain data clearly show that the malicious activity exhibits a unique signature at low frequencies, which is easily differentiable from the spectrum without any malicious activity.

To show the robustness of the detection technique, we ran our test with four different types of Trojans for 5, 10, 20, 40, and 80 s. In our experiments, we collected data of 740 spectra which had no malicious activity and 110 different spectra for each type of Trojan. The experiments were performed at different times of the day to improve confidence in the detection technique for environment changes, such as temperature. The signal stitching technique was used in these experiments to combine data gathered at the different time during the day and therefore with different temperatures. In each of these cases, the Trojans activate randomly, resulting in different time/frequency signatures. The experiments were performed at different times of the day to improve confidence in the fact that there are no false-positives generated by the system. This ensures that potential changes in the environment do not affect the evaluation. As mentioned earlier, even without the presence of malicious activity, a small percentage of the spectrum may be polluted due to harmonics not related to the signal. Therefore, the threshold level for violations is set to 1% of the compared bins of the lower frequency and will flag suspicious activity only if violations exceed the limit.

**Figure 11:**
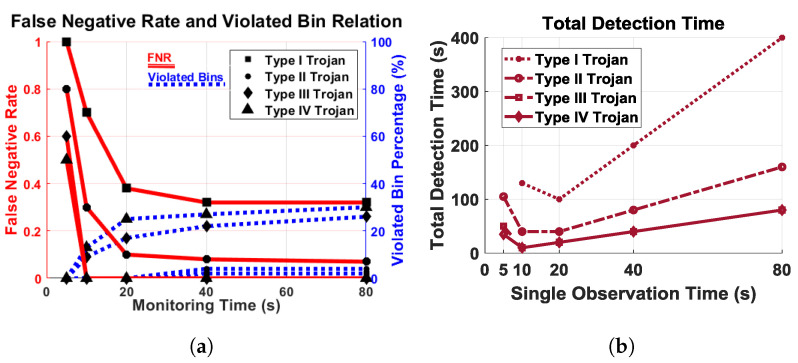
(**a**) Relation of False negative rate (FNR) (red) and minimum number of violated bin (blue) with respect to monitoring time. There are no false positives; (**b**) total time to detect malicious activity with respect to single observation time.

As seen in Figure 11a, the red lines show a false negative rate of Trojans, and the blue lines depict the minimum violated bin percentage. The percentage of minimum violated bin is flat if we go beyond 20 s of monitoring time. The number of bins over the self-referenced threshold does not linearly increase with monitoring time. Figure 11b plots the total detection time with respect to the duration of a single observation to evaluate the cost of testing, based on the number of tests required to detect unauthorized activity. In order to make sure the system is secure, the confidence level is set to 99% for completely automated malicious activity detection. The detection time decreases up to 20 s of observation, but increases after that.

Based on the observation, we see that Type III and IV Trojans can be detected by a single test if the monitoring time is 10 s or more. The Type I Trojans require five repetitions, and Type II Trojans require only two repetitions of 20 s or more to detect, as seen in Figure 11b. Based on our experimental outcome, we decided on a monitoring time of 20 s, which is optimal for all types of Trojans under consideration.

**Figure 12:**
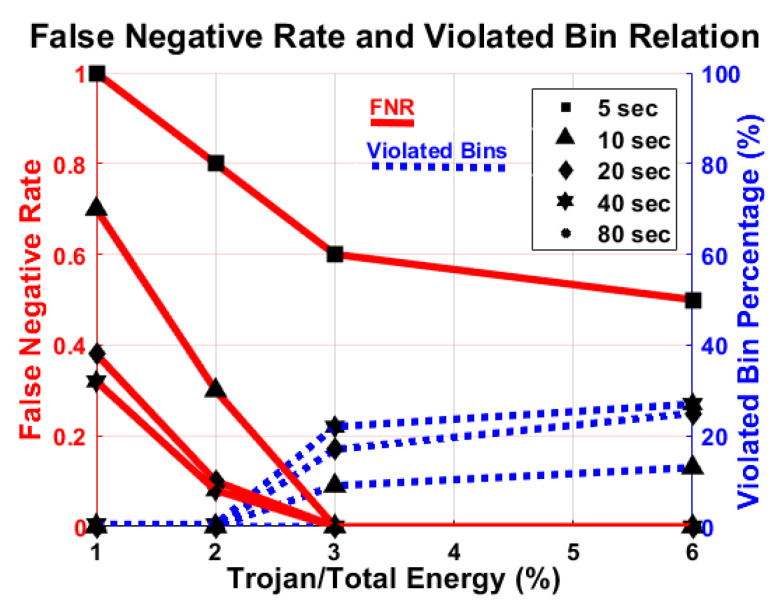
Relation of false negative rate (FNR) (red) and the minimum number of violated bin (blue) with respect to Trojan energy. There are no false positives.

Power consumption by malicious activity is minimal compared to the rest of the activity of the original system. In particular, we focus on determining the smallest detectable Trojan, i.e., the lowest energy that a Trojan may have and still be detected, using one of these four types of malicious activities that are randomly enabled with various activity duration. We run the Trojans on a standalone basis 1000 times and take the average of the total determined energy to calculate the energy of the Trojans. As seen in Figure 12, the false negative rate is drastically reduced when the Trojan energy increased from 1% to 2%, and we reliably detect the Trojan if it is 3% or more of the total energy of the system. In addition, the percentage of violated bins significantly increases when the Trojan energy increases from 2% to 3% but does not change much after that.

### 5.6. Wi-Fi Application

The Wi-Fi application C++ firmware code runs on MpSoC (Odroid-XU3). The foreground application reads sensor information and transmits the data over Wi-Fi to a target destination. To model the malicious activity, we employ a simple firmware Trojan that copies the sensor data to a separate location at random instances. The Odroid board is connected to a low voltage power monitor tool from Monsoon Solution Inc. [53]. The raw power data are collected with PowerTool software for further analysis. The foreground application reads sensor information and transmits the data over Wi-Fi to a target destination. To model the malicious activity, we employ a simple firmware Trojan that copies the sensor data to a separate location at random instances. We assume that the Trojan is always active and do not rely on a particular trigger mechanism.

**Figure 13:**
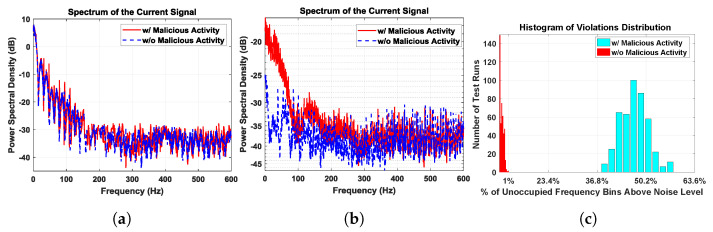
(**a**) Residual spectrum of random WiFi activity signatures with and without malicious activity are identical; (**b**) residual spectrum of Periodic WiFi activity clearly reveals that there is a significant difference; (**c**) histogram of number of spectrum violations for 1000 residual spectra out of which 500 had malicious activity with ***minimum 36.97%*** of spectrum violation, while without malicious activity experiments spectra with ***maximum 0.95%*** of spectrum violation as seen in Table 3.

First, we randomize the patterns of the Wi-Fi application just like the firmware Trojan. Figure 13a shows the residual power pattern (after averaging) under this scenario. Since there is no repeating pattern to the foreground application, the signatures with and without Trojan are identical. Next, we repeat the Wi-Fi application with the same frame, whereas the Trojan is unaltered. Figure 13b shows the residual spectrum of the remainder signal after averaging and filtering. Due to the repetitive nature of the foreground signal, it can be referenced with respect to itself, leaving only small variations due to noise and other factors. The malicious activity due to the Trojan is observable under this scenario. Note that, for detection, we do not need to compare the spectrum with a golden signature; the expectation is to have a flat spectrum, regardless of the power levels. We only need to analyze the spectrum of the measured signal to deduce whether it contains only noise or if there is unwanted activity in addition to the primary circuit current signal and noise.

We performed 500 different experiments, with and without malicious activity. For each experiment, the IoT device is driven into a periodic steady state for approximately 30 s by sending repetitive Wi-Fi messages. We apply the noise threshold explained in Section 4, and count the number of frequency bins above the noise level, which are referred to as violations. The histogram of the number of violations is depicted in Figure 13c. We can observe that the spectrum without malicious activity had a significantly lower number of violations as expected. In fact, there is a very clear separation between the histograms of spectrum violations with and without malicious activity.

Table 3 shows the number of violations for five data sets as a percentage of the number of frequency bins. We set a threshold of 1% violations due to the random nature of noise. In Figure 13c, we can clearly classify all the spectra with less than 1% violations as Trojan free.

## 6. Results and Discussion

This paper presented a malicious Trojan activity detection technique using noise-based self-referencing. The proposed approach utilizes a signal stitching technique to reduce test time by a factor of 25 and analysis computation by more than four times with an optimized recursive FFT. Our self-referencing technique uses power/current consumption measurements without requiring a reliable golden reference. Self-referencing is crucial because it is extremely challenging and, in some circumstances, impossible to attain a golden fingerprint. This novel self-referencing technique is accomplished by placing the design under test in a periodic steady state. The repetitive pattern, through spectral analysis, relies on the primary circuit signal power to a known frequency and identifies malicious behavior.

We also evaluate the sensitivity of our detection method in the presence of measurement and background noise. The proposed approach is evaluated by performing experiments on an IoT device and a commercial SoC with randomly activated and randomly switching Trojans. The experimental results show that the proposed technique can successfully detect malicious activity without causing false alarms. The Trojan detection accuracy depends on the overall energy consumed by the unauthorized activity within an observation bandwidth. This energy may spread over a broad spectrum fall below the measurement sensitivity if the activity level is low. Similarly, if the Trojan is not activated within the test duration, it will not generate a spectral signature. Furthermore, a Trojan that is active for a short burst may also escape detection since its energy will fall below the detectable level.

## Figures and Tables

**Table 1:** Types of Trojans.

Trojan Type	Activity Duration (~ms)	Trojan/Total Energy (%)
Type I	5	1
Type II	10	2
Type III	15	3
Type IV	30	6

**Table 2:** Proposed detection method test modes.

Test Mode	Signal Stitching	Min Monitoring Time (s)	Computation Time (s)	FFT
Mode 1	No	20	60	Regular
Mode 2	Yes	0.8	60	Regular
Mode 3	Yes	0.8	14	Optimized

**Table 3:** Percentage of violations in Data Sets (DS) 1–5.

0.5 % Threshold	w/Trojan (%)			w/outTrojan (%)			False (%)	
	Max	Min	Ave	Max	Min	Ave	Pos.	Neg.
DS1	54.42	36.97	46.99	0.21	0.0	0.12	0.0	0.0
DS2	45.87	39.25	42.32	0.12	0.09	0.10	0.0	0.0
DS3	58.38	38.62	49.23	0.38	0.0	0.09	0.0	0.0
DS4	49.48	37.17	42.85	0.95	0.0	0.17	0.0	0.0
DS5	47.50	38.36	43.37	0.21	0.0	0.11	0.0	0.0

## Data Availability

The data that support the findings of this study are available from the corresponding author upon reasonable request.
